# A Single Imaging Modality in the Diagnosis, Severity, and Prognosis of Pulmonary Embolism

**DOI:** 10.1155/2014/470295

**Published:** 2014-12-14

**Authors:** Hadice Selimoglu Sen, Özlem Abakay, Mehmet Güli Cetincakmak, Cengizhan Sezgi, Süreyya Yilmaz, Melike Demir, Mahsuk Taylan, Hatice Gümüs

**Affiliations:** ^1^Department of Pulmonology, Dicle University Medical Faculty, 21281 Diyarbakir, Turkey; ^2^Department of Radiodiagnostics, Dicle University Medical Faculty, 21281 Diyarbakir, Turkey

## Abstract

*Introduction*. This study aimed to investigate the currency of computerized tomography pulmonary angiography-based parameters as pulmonary artery obstruction index (PAOI), as well as right ventricular diameters for pulmonary embolism (PE) risk evaluation and prediction of mortality and intensive care unit (ICU) requirement. *Materials and Methods*. The study retrospectively enrolled 203 patients hospitalized with acute PE. PAOI was calculated according to Qanadli score. *Results*. Forty-three patients (23.9%) were hospitalized in the ICU. Nineteen patients (10.6%) died during the 30-day follow-up period. The optimal cutoff value of PAOI for PE 30th day mortality and ICU requirement were found as 36.5% in ROC curve analysis. The pulmonary artery systolic pressure had a significant positive correlation with right/left ventricular diameter ratio (*r* = 0.531, *P* < 0.001), PAOI (*r* = 0.296, *P* < 0.001), and pulmonary artery diameter (*r* = 0.659, *P* < 0.001). The patients with PAOI values higher than 36.5% have a 5.7-times increased risk of death. *Conclusion*. PAOI is a fast and promising parameter for risk assessment in patients with acute PE. With greater education of clinicians in this radiological scoring, a rapid assessment for diagnosis, clinical risk evaluation, and prognosis may be possible in emergency services without the need for echocardiography.

## 1. Introduction

The incidence of pulmonary embolism (PE) is about 50 cases in 100,000, according to the European Society of Cardiology 2008 data [1]. There is a large variation in the mortality rates in different clinical severities. Short-term mortality rates ranging from 8 to 58% indicate a potentially fatal disease and emphasize the importance of prognostic factors affecting the disease [2, 3]. The abrupt obstruction of pulmonary blood flow leads to acute pulmonary hypertension and sudden increase in right ventricular afterload [4]. The key to appropriate therapy is risk stratification of patients with PE; the assessment of the haemodynamic status is the cornerstone of this issue [1]. Clinical features, echocardiography, hemodynamic parameters, electrocardiography (ECG), specific biomarkers, and blood gas analysis are tools for acute PE risk evaluation [1, 5]. Patients at high risk require immediate recanalization of the pulmonary arteries and monitorization in the intensive care unit (ICU), whereas patients with low risk may be discharged earlier or take home anticoagulation therapy [1]. However the management strategy in patients with PE at intermediate risk levels is still unclear [1]. The evaluation of right ventricular dysfunction by transthoracic echocardiography (TTE) can be important for predicting early mortality and for guiding treatment modality decision in PE [1, 2, 4, 6]. Previous studies demonstrated an association between echocardiographic parameters and poor in-hospital outcomes in patients with acute PE [7]. Therefore, TTE is a first-line diagnostic test in hemodynamically unstable patients [3, 7]. However, echocardiography is time-consuming and requires experienced personnel, who have limited round-the-clock availability in many emergency departments. Right ventricular image quality may be poor by a transthoracic approach, also limiting TTE as a diagnostic tool [7]. Recently, computerized tomography pulmonary angiography (CTPA) has been frequently used for diagnosing patients with PE suspicion [8]. Computerized tomography pulmonary angiography can clearly illustrate intraluminal clots from main to subsegmental arteries and allows the visualization and measurement of the heart chambers [6]. There is a good correlation between computerized tomography- (CT-) derived and TTE-derived signs of right ventricular dysfunction in previous studies [9, 10]. Currently the risk stratification models based on hemodynamic status and cardiac contagion have been replacing models based on the burden of embolic obstruction of CTPA [1].

The purpose of this study is to investigate the prognostic validity of CTPA-derived image findings with respect to the necessity of therapy in the ICU and 30th day mortality in patients with PE. Therefore, we hypothesized that CTPA may be a valuable rapid and single method of identifying clinical severity and predicting poor clinical outcomes compared to TTE in the same patient groups. CTPA- and TTE-based measurements have been assessed for their prognostic currency in this study.

## 2. Materials and Methods

### 2.1. Baseline Demographic Characteristics and Study Design

The study retrospectively enrolled 203 consecutive patients who were diagnosed and hospitalized with acute PE in the chest disease clinic between June 2011 and June 2013. The study was performed at a tertiary care university hospital. This hospital is a primary referral center for patients with suspected PE. This retrospective cohort study was approved by the local ethics committee. Informed consent was waived because the study was retrospective. One hundred eighty subjects, for whom TTE was performed with pulmonary artery systolic pressure (PASP) assessment within 48 hours of CTPA providing acceptable visualization of the pulmonary arteries, were included in the study cohort. For subjects who underwent multiple TTE examinations within 48 hours of the CTPA, the TTE closest in time to the CT scan was chosen for further evaluation. Information on patient demographics, comorbidities, risk factors for PE, systemic arterial blood pressure (SABP), heart rate, the presence of deep vein thrombosis (DVT), and ICU admissions was obtained from the hospital electronic database system, ICU records, and the patient case notes. Severity of PE according to European Society of Cardiology guidelines was classified in three groups: high risk (patients with shock or hypotension), intermediate risk (presence of right ventricular dysfunction (RVD) or positive myocardial injury marker), and low risk (absence of RVD or negative myocardial injury marker) [1]. Indication for performing CTPA was based on positive results of clinical investigation (determined by revised Wells' score), abnormal findings of laboratory tests (blood gas analysis, D-dimer level, troponin I, and brain natriuretic peptide [BNP]), abnormal results of echocardiography/electrocardiogram indicative of acute right heart dysfunction, abnormal findings of lower limb ultrasound, and results of conventional radiographs suggesting PE.

### 2.2. The CTPA Studies

The CTPA protocol used in the study was the standard PE protocol used in our institution. All patients underwent CTPA on a 64-MDCT scanner (Brilliance CT scanner, Philips Healthcare). All patients were placed in a supine position for scanning, and craniocaudal scanning of the chest was performed. The scanning parameters included a 64 × 0.625 mm collimation, 1 mm slice thickness, 0.5 mm reconstruction increment, and 0.5-second rotation time. One hundred milliliters of a nonionic contrast medium (iohexol [Omnipaque 350, GE Health-care], iopromide [Ultravist 370, Bayer HealthCare, Germany]) was administered at a flow rate of 4-5 mL/s, followed by 40 mL NaCl bolus via an antecubital vein. An automatic injector (CT Injector Missouri, Ulrich Medical) was used for injection of the contrast medium and normal saline. The thorax scanning time was approximately 4-5 seconds, and the time for contrast administration was 25 seconds. The helical CT criterion used to diagnose PE consisted of direct visualization of nonocclusive endoluminal thrombus (central filling defect completely or partially outlined by contrast agent) or of complete occlusion by thrombus in normal-sized or enlarged vessels [11]. All CTPA images were identified and transferred from picture archiving and communication system (PACS) of hospital. Only studies providing acceptable visualization of the pulmonary arteries were included; studies affected by poor vessel enhancement, motion (i.e., respiratory or pulsation) artifacts, and noise were excluded. CT scans were electronically reviewed by two independent observers who were blinded to the clinical history (MGC, HG). Each observer scored the CTPA parameters defined below.

### 2.3. CTPA Studies Were Assessed for the Following Parameters

1. The pulmonary artery obstruction index (PAOI) was calculated according to Qanadli score (0%–100% obstruction), defined by the number of obstructed segmental arteries and corrected on the basis of the estimated degree of occlusion of each vessel (correction factor: 1 = partial obstruction, 2 = complete obstruction) [11].
2. The diameters (minor axes) of right and left ventricles were measured on the axial CT image of the heart at their widest point in diastole (usually the image showing the atrioventricular valves) between the inner surface of the free wall and the surface of the interventricular septum [12].
3. The diameter of the main pulmonary artery was measured on the transverse image at which the right pulmonary artery is in contiguity with the main pulmonary artery [13].
4. The reflux of contrast medium was judged present when it could be detected in the intrahepatic portion of the IVC [12].
5. The pulmonary infarct was deemed present with the identification of a peripheral wedge-shaped consolidation with central lucency [14].
6. Pleural effusion was deemed present according to images on CTPA.

### 2.4. Echocardiographic Evaluation

All TTE examinations were performed in accordance with the recommendations of the American Society for Echocardiography [15]. Standard 2–5 MHz phased array transducers were used to perform TTE studies. Each patient was examined in the supine position, and the patient's position was adjusted to the acoustic window being utilized. Right ventricular dysfunction was defined as echocardiographically measured PASP value >30 mmHg [16]. The patients were divided into RVD (−) and RVD (+) groups based on this criterion (PASP ≤ 30 mmHg and >30 mmHg, resp.). If RV wall hypertrophy was present, these signs were considered chronic, and RV strain from acute PE was excluded [15].

### 2.5. Study Outcomes

The primary end point of the study was an adverse 30-day outcome, defined as death from any cause and ICU requirements. Thirtieth day status was checked from a digital National Population Registration System and hospital records.

### 2.6. Statistical Analyses

Data analysis was carried out using statistical software package software (SPSS 15.0; SPSS Inc, Chicago, Illinois). The Kolmogorov-Smirnov test was used to test for a normal distribution of continuous variables. Data characterized by a normal distribution were expressed as mean and standard deviation. Parameters without such a distribution were expressed as a median with range. Student's *t*-test (normal distribution) or Mann-Whitney (nonnormal distribution) test was used for comparing the two groups. The relationship between the categorical variables was determined using the chi-square test. Pearson correlation analysis and Spearman rank order were used according to distribution of variables. Investigation for a prognostic cutoff value was based on receiver-operating characteristic (ROC) curves. The areas under the curve (AUCs) were calculated. *P* values <0.05 were considered statistically significant. We chose to dichotomize age into categories of older than 60 years and 60 years or younger. Pulmonary artery diameter (PAd) was classified as higher than 30 mm and 30 mm or lower because PAd greater than 30 mm indicates a PA pressure greater than 20 mmHg [17]. The study used logistic regression to assess for an independent association between potential predictors of 30th day mortality and ICU requirement. Regression coefficients and odds ratios were calculated and 95% confidence intervals were given.

*P* values <0.05 in univariate analysis were used as selection criteria for inclusion in the multivariate model. The One Way ANOVA (normal distribution) test or Kruskal-Wallis (nonnormal distribution) test was used for comparing more than two groups. Survival analysis was carried out with the Kaplan-Meier analysis with log-rank test.

## 3. Results

The study group consisted of 180 patients; mean age was 58.33 ± 18.28 years (range: 16–93). The study included 71 (39.4%) males and 109 (60.6%) females. The baseline clinical characteristics, risk factors, arterial blood gas values, and symptoms of the 180 study patients are given in Table 1.

### 3.1. Outcomes

Forty-three patients (23.9%) were hospitalized in the ICU. Nineteen patients (10.6%) died during the 30-day follow-up period.

### 3.2. Clinical Severity

**Figure 1:**
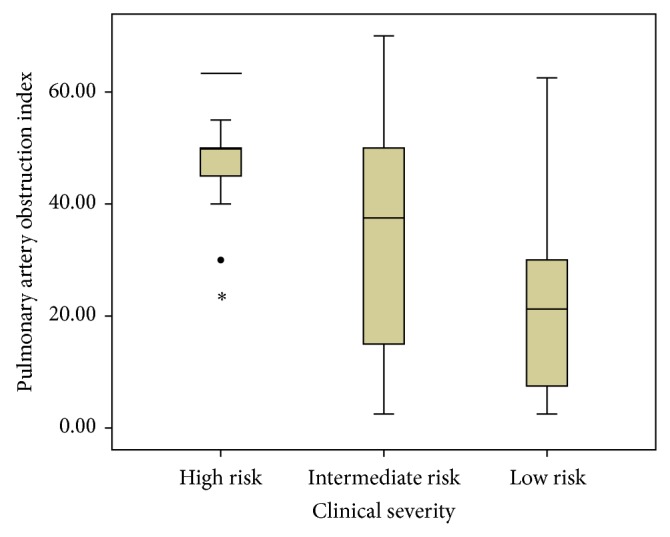
Pulmonary artery obstruction index, among pulmonary embolism patients with different clinical severity.

High risk PE was detected in 15 (8.3%) patients, intermediate risk PE in 119 (66.1%) patients, and low risk PE in 46 (25.6%) patients. Comparisons of mean PAOI values according to clinical severity are presented in Table 2 and Figure 1. The difference between groups was significant.

### 3.3. Receiver Operating Characteristic (ROC) Curve Analysis for Prediction of PAOI Cutoff Values

**Figure 2:**
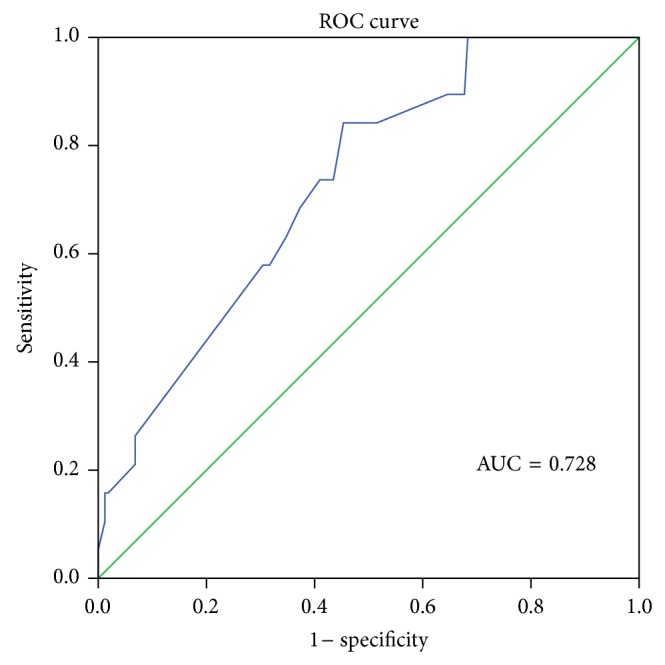
Receiver operating characteristic (ROC) curve of pulmonary artery obstruction index for 30th day mortality; AUC = area under the ROC curve.

**Figure 3:**
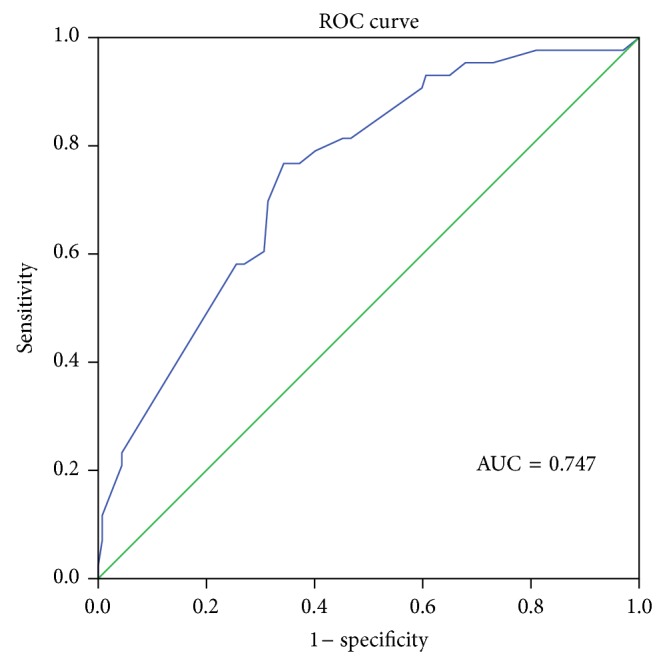
Receiver operating characteristic (ROC) curve of pulmonary artery obstruction index for intensive care unit requirement; AUC = area under the ROC curve.

**Figure 4:**
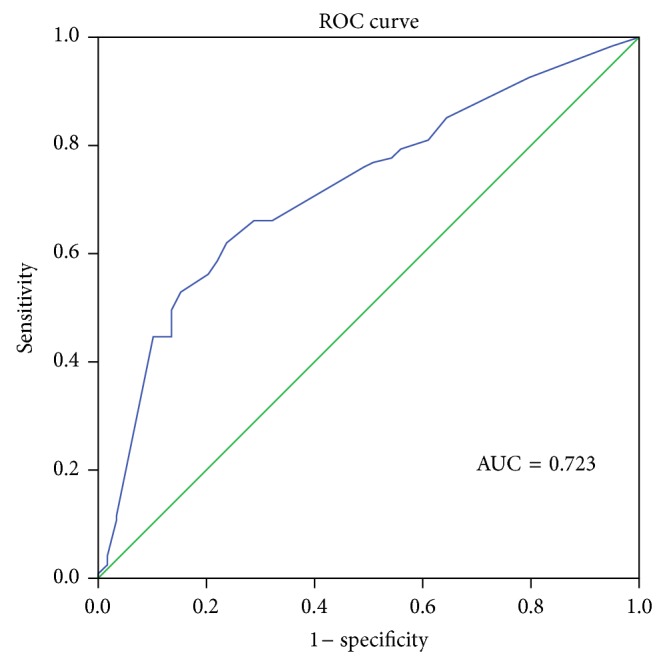
Receiver operating characteristic (ROC) curve of pulmonary artery obstruction index for right ventricular dysfunction; AUC = area under the ROC curve.

The optimal cutoff value of PAOI for PE 30th day mortality and ICU requirement was 36.5% in ROC curve analysis. The sensitivity (sens) and specificity (spec) values were 71.4% sens, 59.1% spec and 76.7% sens, 65.7% spec, for 30th day mortality and ICU requirement, respectively. The areas under the ROC curves (AUCs) were 0.728 and 0.747, respectively (Figures 2 and 3). The optimal cutoff value of PAOI for RVD was 23.75% with 73.9% sens and 52.2% spec in ROC analysis. The area under the ROC curve was 0.723 (Figure 4).

### 3.4. Echocardiographic Evaluation

Forty-six patients were included in the RVD (−) group and 134 patients in RVD (+) group. The mean systolic arterial blood pressure (SABP), pulmonary artery systolic pressure (PASP), PAOI, right ventricular/left ventricular (RV/LV) dimension ratio, and pulmonary artery diameter (PAd) were significantly higher in RVD (+) group. The ICU admission and 30th day mortality ratios were also higher in RVD (+) group. The results are shown in Table 3. The correlation analysis was done between PASP in TTE, RV/LV dimension ratio in CTPA, PAOI and PAd (mm). The pulmonary artery systolic pressure had a significant positive correlation with RV/LV dimension ratio (*r* = 0.531, *P* < 0.001), PAOI (*r* = 0.296, *P* < 0.001), and PAd (*r* = 0.659, *P* < 0.001) (Table 4).

### 3.5. Symptoms and PAOI Values

Mean PAOI values were compared between patients with and without dyspnea, chest pain, hemoptysis, syncope symptoms, and hypoxemia (partial  oxygen  pressure < 60 mmHg) in arterial blood gas analysis. The mean PAOI values were significantly higher in patients with syncope and hypoxemia (Table 5).

### 3.6. Predictors of Mortality in Univariate and Multivariate Analyses

Ages over 60 years, additional disease, presence of malignancy, PAd > 30 mm, PAOI ≥ 36.5%, and RV/LV  dimension  ratio > 1 cm in CT were significantly associated with PE-related 30th day mortality in univariate logistic regression analysis (Table 6). However only being older than 60 years, presence of malignancy, and PAOI ≥ 36.5% were significantly associated with PE-related 30th day mortality on multivariate logistic regression model (Table 7).

### 3.7. Necessity for ICU Treatment

Univariate analysis showed statistically significant results for ages older than 60 years, high risk PE, additional disease, syncope, hypoxemia (PO_2_ < 60 mmHg), PAd > 30 mm, PAOI > 36.5%, and RV/LV > 1 cm in CTPA (Table 8). In the multivariate analysis, only ages over 60 years, syncope, and PAOI > 36.50% were determined as independent predictors of ICU requirement (Table 9).

### 3.8. Kaplan-Meier Estimator

**Figure 5:**
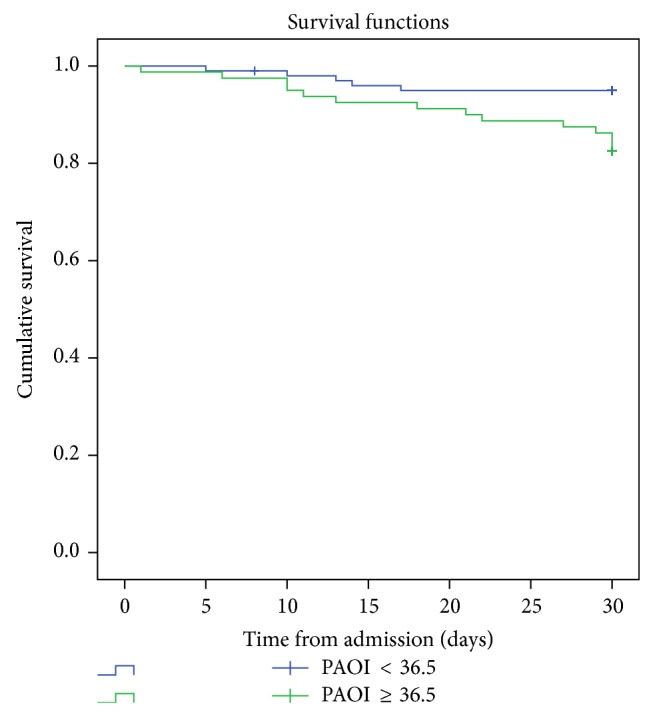
Kaplan-Meier survival analysis of 30th day mortality for patients with pulmonary embolism according to the cutoff values of pulmonary artery obstruction index (PAOI) (*P* = 0.003).

The cutoff value of PAOI obtained by ROC analysis showed a significant survival difference for PE 30th day all-cause mortality in Kaplan-Meier survival analysis (*P* = 0.003, Figure 5).

## 4. Discussion

The mortality rates and necessity of ICU treatment have been investigated in patients with PE in past studies [10, 18]. The results of this study showed that there is a significant correlation between PAOI values and right heart strain in echocardiography. PAOI values increased from the low risk group to the high risk group and the differences were statistically significant. The PAOI values were assessed for the PE 30th day mortality and ICU requirements of patients with PE. PAOI has come to the forefront as an important factor in both univariate and multivariate analyses of these two situations. In addition, PAOI values were significantly higher in patients with syncope and hypoxemia in arterial blood gases, compared to the others.

The adverse results of acute PE are primarily hemodynamic, and a rapid and specific diagnosis is required after admission as death may occur within the first hours of admission [1, 3, 4]. Although TTE is a poor diagnostic test, it is remarkable for risk stratification and guiding treatment strategies in PE [7]. TTE allows noninvasive diagnosis of right ventricular dysfunction (RVD) at the bedside, permits visualization of thrombus particles in the right heart chambers or in the central PA, and is useful for differential diagnosis of cardiac diseases [3, 4]. A correlation between echocardiographic RVD and clinical outcome is clear [2, 3]. The number (percentage) of dead patients in RVD (+) group was also higher in our study. The difference of mortality rates was statistically significant (*P* = 0.004).

The introduction of CTPA in the 1990s as an alternative to ventilation/perfusion scanning has substantially modified the diagnostic approach of acute PE [19]. In concurrence with the increasing number of detectors, the accuracy of CTPA in the diagnosis of PE has increased in the last 20 years. Recently, the PIOPED II study showed that CTPA has a sensitivity of 83% and specificity of 96% for the detection of PE [8]. Today CTPA is widely accepted as the first-line of diagnostic strategy in patients suspected of PE [1]. The one breath hold drawing, full chest submillimetric evaluation, efficacy for differential diagnosis of nonembolic thoracic diseases are advantages of CTPA and have raised this technique as a gold standard for diagnosis of PE. CTPA provides information about cardiac morphology and measurement of the heart chambers and has the potential to provide an alternative to TTE for the assessment of RV function in patients with acute PE [6, 13, 18]. This technique is more rapidly accessible in emergency settings and is more widely available than echocardiography. The screening of the heart chambers at CTPA allows for an evaluation of the right ventricle overload through RV diameters, RV/LV ratio, or the interventricular septal bowing [20]. Many studies have found an association between 30th day mortality after acute PE and CTPA findings of increased embolic burden [18, 21]. The RV/LV diameter ratio at CTPA has a greater accuracy when compared to TTE for the assessment of RVD in patients with acute PE [22]. Quiroz et al. reported that ventricular CT measurements obtained from a four-chamber view were closer to echocardiographic values with similar predictive values for adverse clinical outcomes [18]. Recently Stein et al. reported that cardiac measurements obtained on axial images were comparable with those obtained on four-chamber view reconstructed images [23]. The different cutoffs, sensitivities, and specificities have been reported regarding CT-based signs of RVD. RV/LV diameter ratio cutoff values range from 0.9 to 1.5 on CTPA [24]. An RV/LV diameter ratio greater than 0.9 on CTPA was shown to have a 100% negative predictive value for PE-related mortality and acceptable for predicting the adverse clinical events [18, 25]. George et al. reported that both the increased RV/LV diameter ratio on CTPA and RVD on echocardiography are significant predictors of PE-related short-term mortality with similar prognostic significance [26]. Araoz et al. reported that RV/LV ratio of >1 is associated with 3.6-fold increased risk of admission to ICU [27].

Contrary to these studies, the PROTECT study reported that CTPA-assessed RVD at the time of acute PE diagnosis does not predict all-cause death or a complicated course in normotensive patients [28]. The study found an association between RVD on CTPA and PE-related mortality and hemodynamic collapse within 30 days of PE diagnosis, but there is not a statistical significance [28]. The results of this study do not suggest using RVD on CTPA for the treatment choice decision of acute PE [28]. Similarly CTPA- and TTE-based RV measurements did not predict the incidence of PE-related death in univariate analysis in a prospective study designed by Ozsu et al. [29]. The number of PE-related deaths was limited in this cohort, which may affect the results of study [29]. A recent systematic review and meta-analysis reported that CT-derived interventricular septal bowing and RV/LV diameter ratio are not independent risk factors for long-term death after PE [30]. In our study, right ventricular dysfunction was assessed at CTPA using two-dimensional axial transverse images. The RV/LV diameter ratio on CTPA was significantly higher in echocardiographic RVD (+) group (*P* < 0.001). There is a positive correlation between pulmonary artery systolic pressure (PASP) in TTE and RV/LV diameter ratio on CTPA. An RV/LV  diameter  ratio > 1 cm on CTPA was a predictor of mortality and ICU requirement in univariate analysis; however, it was not achieved to significant values in multivariate analysis in this study.

The widespread use of CTPA for the diagnosis of acute PE has renewed interest in the burden of embolic obstruction in patients with PE [8]. This technique provides the noninvasive direct visualization of emboli in the bilateral main, lobar, and segmental branches of pulmonary arteries [8]. Bankier et al. have applied two pulmonary angiographic indexes, the Miller index, and the Walsh scores, to helical CT to quantify the severity of pulmonary obstruction [31–33]. There was an excellent correlation between both scores and a good interobserver agreement in this study [33]. The CTPA scores suggested by Mastora at al. [34] and Qanadli and coworkers [11] have gained the broadest attention. However, whether pulmonary artery obstruction scores are predictors of RV failure and short-term clinical outcome is controversial [11–13, 21]. Some investigators reported CTPA-derived PA obstruction scores as predictors of severity of PE or PE-related mortality, but others did not find significant predictive value [6, 21]. These different reports are probably due to differences of PE severity among different study populations. The specific PAOI designed by Qanadli et al. is simple and reproducible and has been confirmed in more previous studies about RVD and prognostic evaluation of PE [11–13]. A PAOI of 40% or greater will identify more than 90% of patients with right ventricular dilatation, and a PAOI of less than 40% would be unlikely with acute right ventricular dysfunction in patients with PE [11]. van der Meer et al. reported that PE patients with a PAOI of 40% or higher carry 11.2-fold increased risk of death [13]. Contrary to these studies, three previous studies noted that PAOI can be a legend of the severity of PE episode or of treatment success, but they cannot be used as a predictor of RV failure and death [12, 21, 27].

CTPA imaging findings predictive of mortality have been sought to identify patients who might benefit from more aggressive intervention [35]. This will be helpful for management strategies in patients with PE at intermediate risk. The reperfusion therapy may be considered in selected patients with RV dysfunction and high obstruction scores. High PAOI values were found to be a predictor of RVD, intensive care requirement, and 30th day mortality in our study too. The patients with PAOI values higher than 36.5% carry a 5.7-fold increased risk of death.

The inferior vena cava reflux (IVCR) has recently been described as a predictor of mortality in patients with severe PE [21, 36]. This image is an indirect sign of tricuspid valve insufficiency and frequently may develop as a result of RV dilatation and reduction in the RV output [21, 36]. Ghaye et al. reported a significant correlation between PE mortality and IVCR [21]. Conversely, in the analysis by Collomb et al. there were no significant differences between percentage of patients with IVCR in severe and nonsevere PE groups [12]. Our study also found that IVCR was not a predictor of 30th day mortality and ICU requirement in patients with PE. The percentage of patients with IVCR was similar to patients with and without RVD in echocardiography (*P* = 0.608).

The pulmonary artery diameter (PAd) enlargement in CTPA is another measurement that may serve as an increased pulmonary pressure indicator [36]. A pulmonary artery (PA) diameter greater than 30 mm indicates a PA pressure greater than 20 mmHg [17]. Collomb et al. showed that the diameter of the main PA was significantly higher in patients with high risk PE compared to the patients with low risk PE [12]. The diameter of the main PA and the ratio of the diameters of the main PA and the aorta were not an indicator of mortality or severity of acute PE in three other studies [13, 21, 27], which was supported by our study that PAd was significantly higher in patients with RVD and high risk PE. PAd greater than 30 mm was found as a prognostic factor and a marker for ICU requirement in univariate analysis. However PAd was not achieved to significant values in multivariate analysis.

The incidence of lung infarcts was documented to be up to 50% and 36% in different studies [37, 38]. Lung infarctions have been identified to be associated with significantly lower mortality rates both during initial therapy and after discharge [37]. In a previous study, lung infarcts were not correlated with mortality, which might be due to the small sample size [38]. Similarly lung infarctions were not associated with RVD in echocardiography, 30th day mortality, and ICU requirement in our study.

It was stated that the pleural effusion secondary to pulmonary embolism could be either a transudate or an exudate. The pathogenetic mechanism responsible for transudates was considered to be an increase of the systemic venous pressure at the parietal pleural surface secondary to pulmonary hypertension and increases in the right ventricular pressure [39]. In the study of Findik et al. [39], multidetector CT revealed pleural effusions in 79% of the patients with massive pulmonary embolism. There was not any difference in percentages of pleural effusion existence between survivors and survivors in a study by Furlan et al. [20]. Pleural effusion was not found to be a predictor of mortality and ICU requirement in our study.

## 5. Conclusion

Risk stratification of patients with PE is important because optimal management, monitoring, and therapeutic strategies depend on the prognosis. Recent studies have demonstrated that CTPA not only allows diagnosis of PE but also enables accurate assessment of PE severity in a single examination. Nevertheless, our data show that increased embolic burden is associated with PE severity, 30th day short-term mortality, and ICU hospitalization necessity. Pulmonary artery obstruction index is a fast and promising parameter for risk assessment in patients with acute pulmonary embolism. With greater education of clinicians in this radiological scoring, a rapid assessment for diagnosis, clinical risk evaluation, and prognosis may be possible in the emergency services without the need for TTE. Many CT findings that may allow refinement of the risk stratification are still under evaluation. Our study was limited by its retrospective character. According to new large prospective studies, CTPA perhaps will be a single imaging modality for PE in the future.

## Figures and Tables

**Table 1:** Baseline characteristics of patients with pulmonary embolism (*n* = 180).

	*n*	%
Gender		
Male	71	39.4
Female	109	60.6
SBP < 90 mmHg	18	10.0
SO_2_ < 90%	78	43.3
PaO_2_ < 60 mmHg	70	38.9
Deep vein thrombosis	83	46.1
Additional disease	86	47.8
CCD	41	22.7
CPD	11	6.1
DM	9	5.0
Others	25	13.8
Clinical risk factors		
Puerperium	10	5.5
Surgery	61	33.9
Trauma	25	13.9
Malignancy	18	10
Immobilization	24	13.3
Others	25	13.8
Idiopathic VTE	17	9.4
ICU requirement	43	23.9

*n*: number, SBP: systolic blood pressure, SO_2_: arterial oxygen saturation, PaO_2_: partial oxygen pressure, CCD: chronic cardiovascular disease, CPD: chronic pulmonary disease, DM: diabetes mellitus, VTE: venous thromboembolism, and ICU: intensive care unit.

**Table 2:** Comparison of mean PAOI values according to clinical severity.

Clinical severity	Mean difference	Std. error	95% CI	*P* value
High-intermediate	13.91	3.04	6.15–21.66	**<0.001**
High-low	26.24	3.36	17.82–34.67	**<0.001**
Intermediate-low	12.33	2.73	5.69–18.96	**<0.001**

**Table 3:** Comparison of CT findings and echocardiographic right ventricular dysfunction.

	RV dysfunction (+) PASP > 30 mmHg	RV dysfunction (−) PASP ≤ 30 mmHg	*P* value
SABP	109.78 ± 15.00	119.89 ± 6.70	** <0.001**
PASP^*^	50.22 ± 14.35	23.21 ± 2.11	**<0.001**
PAOI^*^	34.97 ± 12.82	21.08 ± 9.76	**<0.001**
RV/LV diameter^*^	1.27 ± 0.37	0.85 ± 0.18	**<0.001**
PAd^*^	29.23 ± 5.28	23.21 ± 2.50	**<0.001**
Pulmonary infarct^&^	27 (20.14%)	14 (30.43%)	0.159
Pleural effusion^&^	51 (38.05%)	17 (36.95%)	1.00
IVC reflux^&^	65 (48.50%)	25 (54.34%)	0.608
ICU admission^&^	42 (31.34%)	1 (2.17%)	**<0.001**
30th day mortality^&^	19 (14.17%)	0 (0%)	**0.004**

^*^Mean ± SD.

^&^Number (percentage) of patients.

SABP: systemic arterial blood pressure, PASP: pulmonary artery systolic pressure, PAOI: pulmonary arterial obstruction index, RV/LV: right ventricular/left ventricular, IVC: inferior vena cava, PAd: pulmonary artery diameter, and ICU: intensive care unit.

**Table 4:** Parameters with correlation between echocardiography (ECHO) and computed tomography pulmonary angiography (CTPA).

	Pearson *r*	*P* value
PASP and RV/LVd	0.531	**<0.001**
PASP and PAOI (%)* *	0.296	**<0.001**
PASP and PAd (mm)	0.659	**<0.001**

PASP: pulmonary artery systolic pressure, RV/LVd: right ventricular/left ventricular dimension in CTPA, PAOI: pulmonary arterial obstruction index, and PAd: pulmonary artery diameter.

**Table 5:** The relation among the mean PAOI values and patient symptoms and presence of hypoxemia in arterial blood gas analysis.

	PAOI (mean ± standard deviation)	*P* value
Dyspnea		
Absent (*n* = 9)	23.88 ± 16.58	0.206
Present (*n* = 171)	31.83 ± 18.44	
Chest pain		
Absent (*n* = 35)	29.42 ± 17.08	0.469
Present (*n* = 165)	31.94 ± 18.71	
Hemoptysis		
Absent (*n* = 141)	31.47 ± 18.65	0.982
Present (n = 39)	31.39 ± 17.64	
Syncope		
Absent (*n* = 165)	30.55 ± 18.55	**0.029**
Present (*n* = 15)	41.33 ± 13.32	
PaO_2_ < 60 mmHg)		
Absent (*n* = 110)	28.04 ± 18.26	**0.002**
Present (n = 70)	36.81 ± 17.41	

PAOI: pulmonary arterial obstruction index, PaO_2_: partial oxygen pressure.

**Table 6:** Univariate analysis of the possible prognostic factors in patients with pulmonary embolism.

	OR	%95 CI	*P* value
Age > 60 years	**5.536**	1.553–19.737	**0.008**
Male gender	0.884	0.330–2.365	0.806
Massive PE	0.471	0.059–3.750	0.477
Additional disease	**3.461**	1.190–10.064	**0.023**
Syncope	0.430	0.110–1.684	0.225
Hemoptysis	0.651	0.180–2.360	0.514
PO_2_ < 60 mmHg	1.870	0.719–4.863	0.199
Malignancy	**5.731**	1.847–17.778	**0.003**
Trauma	0.706	0.153–3.261	0.656
Operation	0.426	0.563–3.902	0.456
DVT	0.907	0.408–2.742	0.907
PAd > 30 mm	**3.148**	1.197–8.278	**0.020**
PAOI 36.5%	**6.429**	1.803–22.929	**0.004**
RV/LV > 1 cm in CT	**4.315**	1.210–15.389	**0.024**
VCIR	1.125	0.434–2.915	0.808
Pulmonary infarct	1.648	0.456–5.960	0.446
Pleural effusion	0.507	0.195–1.319	0.164

PE: pulmonary embolism, ICU: intensive care unit, sPESI: simplified pulmonary embolism severity index, DVT: deep vein thrombosis, VCIR: vena cava inferior reflux, PAOI: pulmonary artery obstruction index, PAd: pulmonary artery diameter, RV/LV: right ventricular/left ventricular ratio, and PASP: pulmonary artery systolic pressure.

**Table 7:** Multivariate analysis of the possible prognostic factors in patients with pulmonary embolism.

	OR	%95 CI	*P* value
Age > 60 years	**4.854**	1.164–20.240	**0.030**
Additional disease	1.617	0.490–5.341	0.430
Malignancy	**7.746**	1.920–31.955	**0.004**
PAd > 30 mm	0.917	0.255–3.292	0.894
PAOI > 36.5%	**5.657**	1.170–27.355	**0.031**
RV/LV > 1 cm in CT	2.470	0.568–10.749	0.228

PE: pulmonary embolism, PAd: pulmonary artery diameter, PAOI: pulmonary artery obstruction index, and RV/LV: right ventricular/left ventricular ratio.

**Table 8:** Univariate analysis of the possible factors affecting admission to intensive care unit.

	OR	%95 CI	*P* value
Age > 60 years	**4.707**	2.097–10.562	**<0.001**
Male gender	0.884	0.436–1.791	0.731
PE with high risk	**3.765**	1.387–10.216	**0.009**
Additional disease	**2.543**	1.246–5.186	**0.010**
Syncope	**5.779**	1.927–17.357	**0.002**
Hemoptysis	0.944	0.408–2.185	0.893
PaO_2_ < 60 mmHg	**2.499**	1.243–5.025	**0.010**
Malignancy	1.689	0.593–4.810	0.326
Trauma	1.285	0.497–3.322	0.604
Operation	0.511	0.232–1.123	0.095
DVT	0.703	0.350–1.412	0.323
PAd > 30 mm	**4.283**	2.072–8.852	**<0.001**
PAOI > 36.5%	**5.632**	2.505–12.664	**<0.001**
RV/LV > 1 cm in CT	**2.550**	1.189–5.469	**0.016**
VCIR	1.063	0.536–2.110	0.861
Pulmonary infarct	0.720	0.304–1.706	0.456
Pleural effusion	0.849	0.415–1.736	0.654

PE: pulmonary embolism, PaO_2_: partial oxygen pressure, ICU: intensive care unit, sPESI: simplified pulmonary embolism severity index, DVT: deep vein thrombosis, PAd: pulmonary artery diameter, PAOI: pulmonary artery obstruction index, RV/LV: right ventricular/left ventricular ratio, CT: computed tomography, and VCIR: vena cava inferior reflux.

**Table 9:** Multivariate analysis of the possible factors affecting admission to intensive care unit.

	OR	%95 CI	*P* value
Age > 60 years	**5.056**	1.859–13.750	**0.001**
PE with high risk	2.483	0.729–8.462	0.146
Additional disease	1.250	0.535–2.917	0.606
Syncope	**4.474**	1.203–16.632	**0.025**
PaO_2_ < 60 mmHg	1.295	0.565–2.971	0.541
PAd > 30 mm	1.264	0.472–3.389	0.641
PAOI > 36.5%	**3.671**	1.309–10.292	**0.013**
RV/LV > 1 cm in CT	1.319	0.523–3.325	0.557

PE: pulmonary embolism, PaO_2_: partial oxygen pressure, PAd: pulmonary artery diameter, PAOI: pulmonary artery obstruction index, RV/LV: right ventricular/left ventricular ratio, and CT: computed tomography.

## Limitations

Our study has the following limitations. This is a retrospective study. There could be potential selection bias, with TTE being performed in those with more severe PE (i.e., a high fraction of central PE) and higher likelihood of RV dysfunction. We did not exclude patients with other preexisting diseases such as chronic obstructive pulmonary disease, which can also lead to abnormal RV enlargement.
